# Genetically predicted tea intake increases the risk of osteoarthritis: A Mendelian randomization study

**DOI:** 10.3389/fgene.2022.1004392

**Published:** 2022-10-04

**Authors:** Gang Li, Zhe Zhang, Yang Liu

**Affiliations:** Department of Sports Medicine, The First Affiliated Hospital of Xinjiang Medical University, Urumqi, Xinjiang, China

**Keywords:** tea, osteoarthritis, Mendelian randomization, risk factor, causal association

## Abstract

**Background:** This study aimed to clarify the relationship between tea consumption and osteoarthritis (OA).

**Methods:** Common single-nucleotide polymorphisms (SNPs) from the Open Genome-wide Association Studies database were obtained. Summary statistics on OA were retrieved from the second dataset that enrolled 50,508 participants (10,083 OA cases) of European ancestry. The causal association between tea intake and OA was tested using two-sample Mendelian randomization (MR) analysis.

**Results:** Tea consumption has adverse effects on OA. (inverse-variance weighted method: OR = 1.19, 95% CI = 1.08–1.30; weighted median method: OR = 1.22, 95% CI = 1.07–1.40). The MR–Egger regression intercept (MR intercept = −0.002; *p* = 0.73) showed no evidence of directional pleiotropy. Moreover, no evidence of underlying heterogeneity in MR analysis was found according to Cochran’s Q test and funnel and forest analyses.

**Conclusion:** A genetically predicted high daily tea intake can increase the risk of OA.

## Background

Osteoarthritis (OA) is the most prevalent type of arthritis and is characterized by articular cartilage degeneration, remodeling of underlying bone, and synovitis (Loeser et al., 2012; Martel-Pelletier et al., 2016). According to previous studies of 291 conditions, hip and knee OA is the 11th highest disease in terms of global disability and 38th highest in disability-adjusted life years (Cross et al., 2014). Epidemiological studies from Prieto-Alhambra et al. (2014) showed that OA is prevalent among aged individuals and severely affects the quality of life. Patients with OA suffer higher risks of disability and all-cause mortality than their healthy counterparts. These burdens motivate researchers to explore underlying mechanisms and develop effective methods against OA (Palazzo et al., 2016).

Accumulating lines of evidence show that OA is a multifactorial disorder of articular cartilage. Aging, obesity, inflammation, trauma, and overloading are closely associated with OA (Kulkarni et al., 2016; Rahmati et al., 2017; Nickien et al., 2018; Berenbaum and Walker, 2020; Wang et al., 2020). Therapies that suppress inflammatory mediators, such as cyclooxygenase-2, and oxidative stress and enhance autophagy, have shown efficacy in animal models and clinical settings to some extent. The elderly may benefit from complementary and alternative medicine, such as tea (Lapane et al., 2013). However, the association and underlying mechanism between tea intake and OA remains inconsistent and needs further investigation.

Tea is a popular drink worldwide. Tea contains chemicals, such as caffeic acid, caffeine, catechin, coumaric acid, and gallic acid, which have effects on OA (Luk et al., 2020). However, few studies have explored the relationship between tea intake and OA in humans, and the findings are inconsistent. In a 5-years cohort study, Takiguchi et al. (2019) reported that low intake of green tea is associated with a high incidence of knee OA in males but not in females. A randomized clinical study revealed that green tea intake could not decrease the pain and stiffness of patients with OA as assessed by the Western Ontario and McMaster Universities Osteoarthritis Index (WOMAC) questionnaire (Hashempur et al., 2018). However, in animal models, tea intake can decrease cartilage degeneration, inhibit inflammation, and function as a protective factor. Further studies are needed to explore their relationship (Luk et al., 2020).

Summary-level datasets were used to verify the relationship between tea intake and OA. Mendelian randomization (MR) was adopted to test their causal links.

## Materials and methods

### Data source for genetic variants

The MRC IEU Open GWAS Project (https://gwas.mrcieu.ac.uk/) is an online database for searching GWAS datasets and traits. SNP information of tea intake was extracted from Neale Lab (http://www.nealelab.is/uk-biobank, GWAS round 2), which consists of more than 3,49,376 samples from European ancestry (Wang et al., 2021). Based on a dietary questionnaire, daily tea consumption was determined at baseline. The touchscreen question that needs to be answered is “How many cups of tea do you drink every day?” (including black and green tea). A total of 2,672 unique single-nucleotide polymorphisms (SNPs) were chosen based on minor frequency >1% of SNPs with strong correlation as *p* < 5 × 10^8^. We clumped the 2,672 SNPs at a 10,000 kb window and linkage disequilibrium with *r* ^*2*^ < 0.001 by using the 1,000 Genomes Project reference for the European samples. The results confirmed the genetic independence among exposure variants. A total of 45 significant SNPs related to exposure were identified. Large-scale GWAS was carried out from the UK Biobank, which included 50,508 European ancestry, and yielded summary-level data with a clinical diagnosis for OA (10,083 cases and 40,425 controls). Protocols related to the data have been released and described in previous studies (Wu et al., 2013; Okada et al., 2014; Zengini et al., 2018). Summary data can be obtained from the UK MRC IEU Open GWAS Project database (http://gwas.mrcieu.au.uk). Informed consent was obtained from all participants. All GWAS-related current analyses were approved by the relevant ethics committees.

### Statistical analysis

MR analysis requires genetically predicted SNP exposure that is not associated with potential confounders (Burgess et al., 2013). In the first step, we selected 45 SNPs that were independently associated with tea consumption. In the second step, we identified the association of each SNP with OA risk. In the third step, MR was used to estimate the uncompounded causal relationship between exposure and outcome risk. Based on summary statistics from two different GWASs, we performed a two-sample MR to estimate the causal effect of exposure on outcomes. We then assessed the causal relation by using GWAS datasets from tea intake and OA with the independent 45 SNPs as instruments (Hartwig et al., 2016). An inverse-variance weighted (IVW) approach based on combining Wald ratio estimates from different SNPs was employed to estimate the causal effect and provide consistent estimates of the effect of genetic variants on outcome when the instrument’s assumptions are verified (Pierce and Burgess, 2013). Although multiple variants in MR analysis increased the statistical power, they might not be valid instrumental variants due to their pleiotropic nature (Hartwig et al., 2016). Therefore, MR–Egger regression and weighted median estimators were used to avoid pleiotropy (multiple genetic variants associated with multiple variables). MR and Egger are weighted regression models that introduce an intercept to account for directional pleiotropy. A horizontal pleiotropy occurs when intercept values differ from zero (Bowden et al., 2015). Weighted linear regression was conducted using MR–Egger to estimate the coefficients of gene exposure and outcome (Bowden et al., 2015). In this method, unbiased estimates are used when pleiotropic instruments are used, in which the pleiotropic effects are unrelated to instrument size (Burgess and Thompson, 2017). Weighted median estimation supports a robust evaluation of causal power, even when genetic variants are invalid instrument factors contributing up to 50% of the explanation (Bowden et al., 2016). In comparison with MR–Egger, weighted median approach maintains greater precision in the estimates. Additionally, mode-based causal estimation consistently estimates true causal effects when most instruments generate consistent MR estimates.

Heterogeneities between SNP exposure were examined by Cochran’s Q statistics and funnel plots, respectively (Egger et al., 1997). Leave-one-out was used to visualize whether the causal relation is influenced by one outlier SNP alone. We also performed Wald ratio estimates on each SNP associated with OA.

All MR analyses were performed using Two-sample MR packages, and statistical significance was tested at *p* < 0.05 (Hemani et al., 2018).

## Results

### Instrumental variables for Mendelian randomization

Forty-five independent SNPs related to genetic tea intake were determined using *R* ^*2*^ statistic. *F*-statistics were achieved to assess the strength of exposure-related instruments (Pierce et al., 2011; Shim et al., 2015). Table 1 provides detailed information about SNPs and exposures associated with selected SNPs.

**TABLE 1:** Characteristics of tea consumption-associated SNPs.

SNP	Position	EAF	EA	Beta	SE	*P*	N	*R* ^*2*^	F-statistic
rs1030510	7:17100273	0.45	G	−0.0436	0.0069	3.6E-10	3,49,376	0.000114	40
rs10741694	11:16286183	0.63	C	0.0404	0.0071	1.53E-08	3,49,376	0.0000927	32
rs11022751	11:13307613	0.27	C	0.0497	0.0078	1.83E-10	3,49,376	0.000116	41
rs112476491	7:17204040	0.03	A	−0.1186	0.0194	8.88E-10	3,49,376	0.000107	37
rs11487328	1:174601659	0.38	C	−0.0493	0.0071	5.16E-12	3,49,376	0.000138	48
rs11636222	15:75515312	0.23	G	−0.0557	0.0089	3.79E-10	3,49,376	0.000112	39
rs12591786	15:60902512	0.16	T	−0.0609	0.0096	2.32E-10	3,49,376	0.000115	40
rs12600469	17:40834073	0.62	T	0.0406	0.0071	1.22E-08	3,49,376	0.0000936	33
rs12901092	15:75374145	0.39	A	−0.0654	0.0071	3.2E-20	3,49,376	0.000243	85
rs12916473	15:75321999	0.04	A	0.1233	0.0185	2.63E-11	3,49,376	0.000127	44
rs140775622	20:62962869	0.17	T	0.0707	0.0099	9.33E-13	3,49,376	0.000146	51
rs1481012	4:89039082	0.11	G	−0.0778	0.0109	9.41E-13	3,49,376	0.000146	51
rs149375687	5:152034989	0.27	T	−0.0449	0.0078	7.26E-09	3,49,376	0.0000948	33
rs1601409	12:17066769	0.46	G	0.0382	0.0069	3.67E-08	3,49,376	0.0000877	31
rs1669433	12:11349732	0.84	G	0.0551	0.0093	3.33E-09	3,49,376	0.0001	35
rs17645813	7:17419697	0.08	A	−0.1058	0.013	3.32E-16	3,49,376	0.00019	66
rs199621380	1:150700614	0.41	G	0.0413	0.007	4.53E-09	3,49,376	0.0000996	35
rs200062544	7:17260246	0.47	A	0.049	0.007	2.64E-12	3,49,376	0.00014	49
rs2315024	19:19423817	0.33	A	0.0434	0.0073	2.98E-09	3,49,376	0.000101	35
rs2465018	6:51241140	0.23	A	0.0635	0.0082	1.38E-14	3,49,376	0.000172	60
rs2472297	15:75027880	0.27	T	0.1576	0.0078	3.82E-91	3,49,376	0.00117	408
rs28548701	15:74346021	0.8	C	−0.0502	0.0086	5.82E-09	3,49,376	0.0000975	34
rs28676340	15:75449794	0.16	G	−0.0564	0.01	1.96E-08	3,49,376	0.000091	32
rs34591452	15:74492585	0.24	T	0.0759	0.0081	5.48E-21	3,49,376	0.000251	88
rs34606716	7:75820449	0.24	A	−0.0453	0.0082	2.7E-08	3,49,376	0.0000873	31
rs3815455	7:75611756	0.29	T	0.0647	0.0076	1.74E-17	3,49,376	0.000207	72
rs397074	15:74599997	0.31	C	−0.0521	0.0075	2.8E-12	3,49,376	0.000138	48
rs4410790	7:17284577	0.63	C	0.1215	0.0072	1.89E-64	3,49,376	0.000814	285
rs4817505	21:34343828	0.39	C	0.0411	0.0071	6.22E-09	3,49,376	0.0000959	34
rs4887165	15:74889356	0.81	C	0.0539	0.0089	1.22E-09	3,49,376	0.000105	37
rs60223362	7:17459648	0.2	C	−0.0747	0.0086	5.35E-18	3,49,376	0.000216	75
rs6495129	15:75196717	0.2	T	−0.0582	0.0086	1.35E-11	3,49,376	0.000131	46
rs6697410	1:26756209	0.74	T	0.0436	0.0079	4.1E-08	3,49,376	0.0000872	30
rs6965666	7:17177312	0.28	C	−0.0503	0.0078	9.14E-11	3,49,376	0.000119	42
rs7174381	15:75613289	0.31	C	0.0522	0.0075	3.85E-12	3,49,376	0.000139	48
rs73071153	7:17545964	0.03	A	−0.1312	0.0194	1.32E-11	3,49,376	0.000131	46
rs73075157	7:17566844	0.13	A	−0.0678	0.0103	5.42E-11	3,49,376	0.000124	43
rs73169830	22:24885208	0.08	C	0.1027	0.0131	3.81E-15	3,49,376	0.000176	61
rs73424602	22:41461176	0.4	T	−0.0432	0.007	7.84E-10	3,49,376	0.000109	38
rs77821156	7:17331450	0.11	G	0.0643	0.0113	1.39E-08	3,49,376	0.0000927	32
rs79217743	15:75117912	0.14	T	−0.0602	0.0102	3.34E-09	3,49,376	0.0000997	35
rs79413667	7:17399486	0.03	G	−0.1171	0.0201	6.03E-09	3,49,376	0.0000971	34
rs79694830	7:17286087	0.06	T	0.0951	0.015	2.26E-10	3,49,376	0.000115	40
rs7999399	13:89233505	0.55	T	0.0379	0.0069	4.96E-08	3,49,376	0.0000863	30
rs9624470	22:24820268	0.58	A	0.0729	0.007	3.06E-25	3,49,376	0.00031	108

SNP, single nucleotide polymorphism; EAF, effect allele frequency; EA, effect allele; SE, standard error. The *R* ^*2*^ was calculated as follows: 2 × beta^2^ × EAF × (1 − EAF)/[2 × beta^2^ × EAF × (1 − EAF) + se^2^ × 2 × N × EAF (1 − EAF)]. The *F*-statistic for each SNP was calculated as follows: *F* = (N − 2) × *R* ^2^/(1 − *R* ^2^).

### Mendelian randomization between tea intake and OA

**FIGURE 1:**
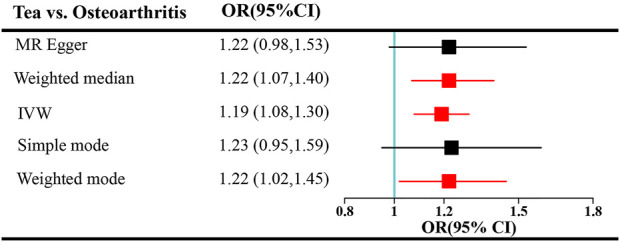
Forest plots of MR study using genetically predicted tea consumption with OA. Inverse-variance weighted methods, MR–Egger analysis, weighted median, and simple and weighted modes were used in this study.

**FIGURE 2:**
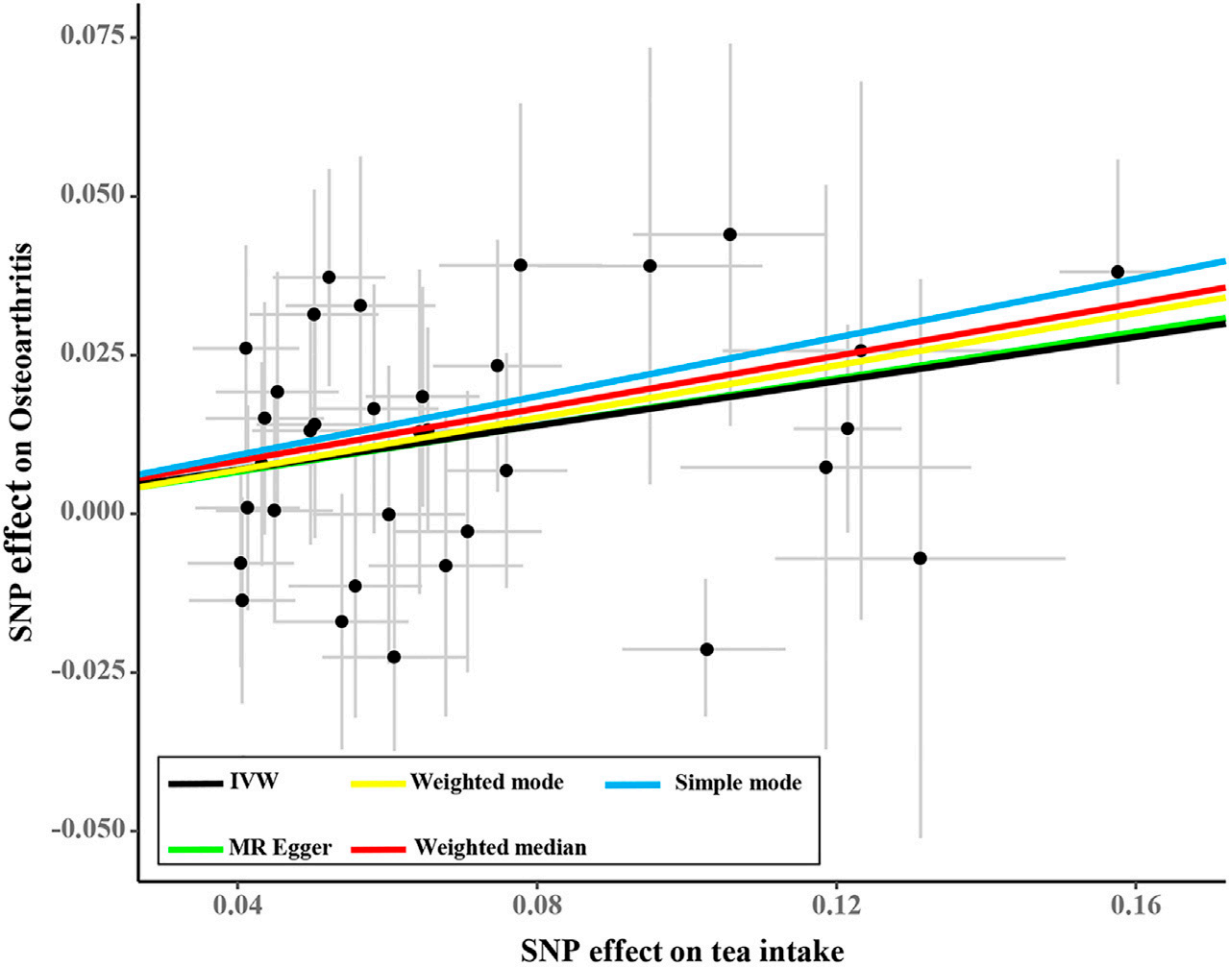
Scatter plot of the effect size for each SNP on tea consumption and OA.

Tea consumption was positively associated with OA according to the IVW approach [odds ratio (OR) = 1.22; 95% confidence interval (CI): 1.08, 1.30; Figures 1, 2]. An intercept represents the average pleiotropic effect across genetic variants (that is, how a variant affects the outcome on average). MR–Egger regression found that directional pleiotropy was unlikely to bias the results. If the intercept is different from zero (MR–Egger test), then directional pleiotropy exists (MR–Egger intercept = −0.002; *p* = 0.73). Tea consumption was not related to OA *via* MR–Egger (OR = 1.22; 95% CI: 0.98, 1.53; Figures 1, 2). However, weighted median (OR = 1.22; 95% CI: 1.07, 1.40) and weighted mode (OR = 1.22; 95% CI: 1.02, 1.45) suggested a causal relation between them (Table 2; Figure 2). As a result, IVW, weighted median, modes, and MR–Egger methods did not present consistent results regarding tea consumption and OA. Compared with the MR–Egger analysis, the weighted median estimator maintains greater precision in estimates, which may suggest a causal association between high tea intake and increased risk of OA (Bowden et al., 2015).

**TABLE 2:** MR estimates from each method of the causal effect of tea consumption on osteoarthritis risk.

MR method	OR	SE	95% CI	*p* value	Cochran’s Q statistic	Heterogeneity *p* value
Inverse variance weighted	1.19	0.04	1.08–1.30	0.0001	35.71	0.70
MR-Egger	1.22	0.11	0.98–1.53	0.077	35.81	0.73
Maximum likelihood method	1.23	0.07	1.06–1.40	0.004	35.64	0.74

MR, Mendelian randomization; SNP, single-nucleotide polymorphism; OR, odds ratio; SE, standard error.

### Heterogeneity and sensitivity tests

**FIGURE 3:**
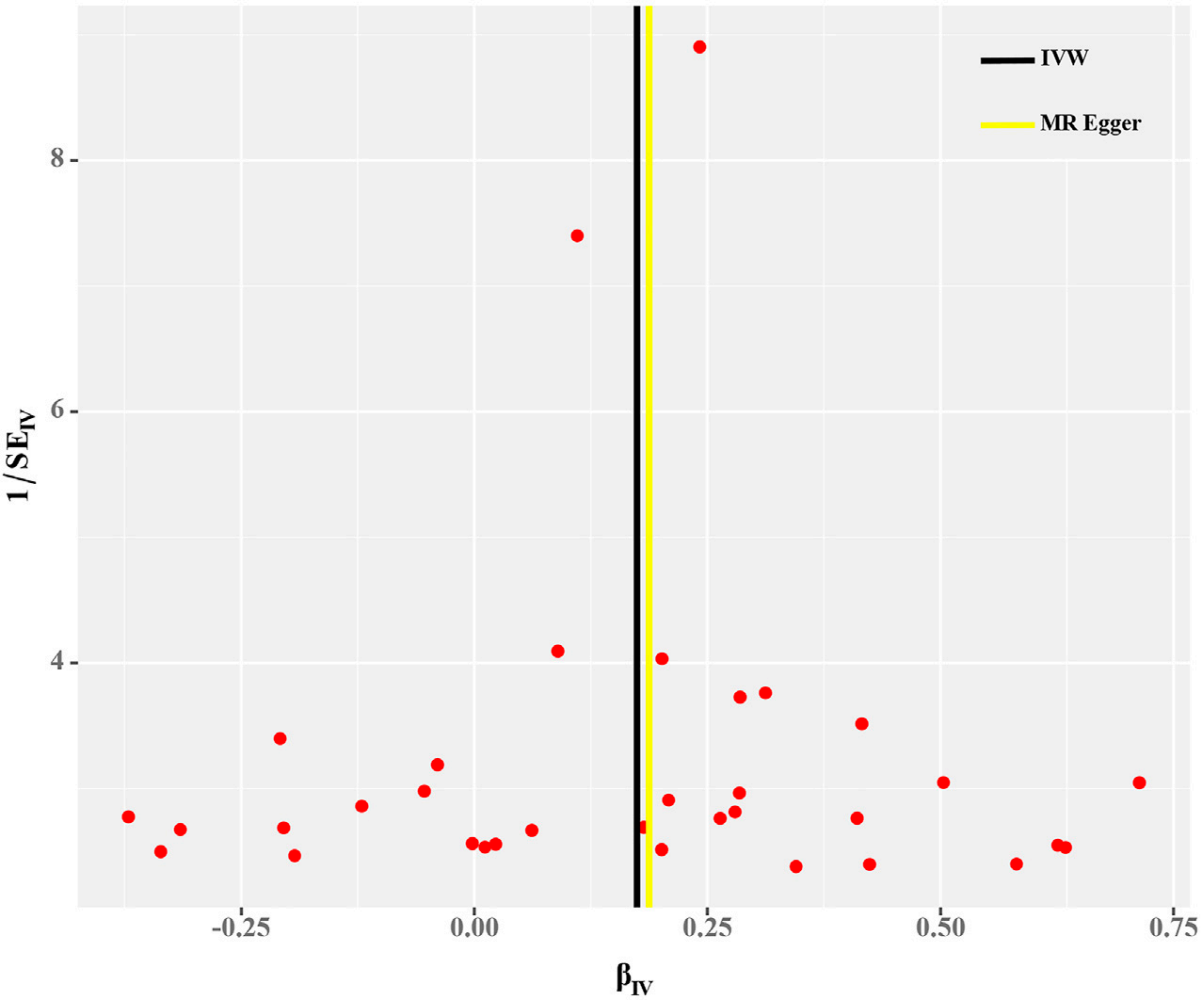
Funnel plot for the causal estimates of each tea SNP with OA.

**FIGURE 4:**
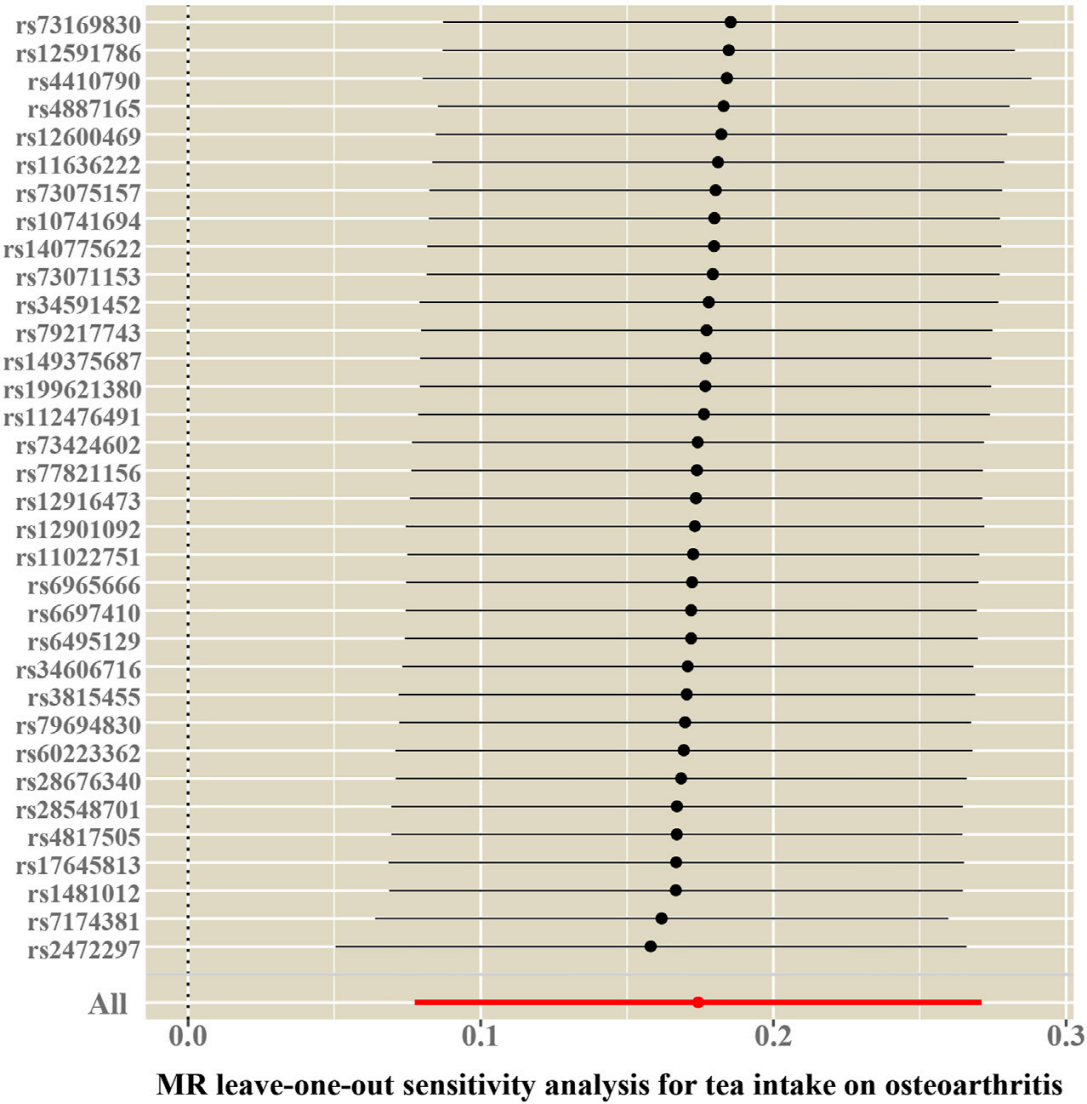
Leave-one-out sensitivity analysis for the causal association between tea consumption and OA.

**FIGURE 5:**
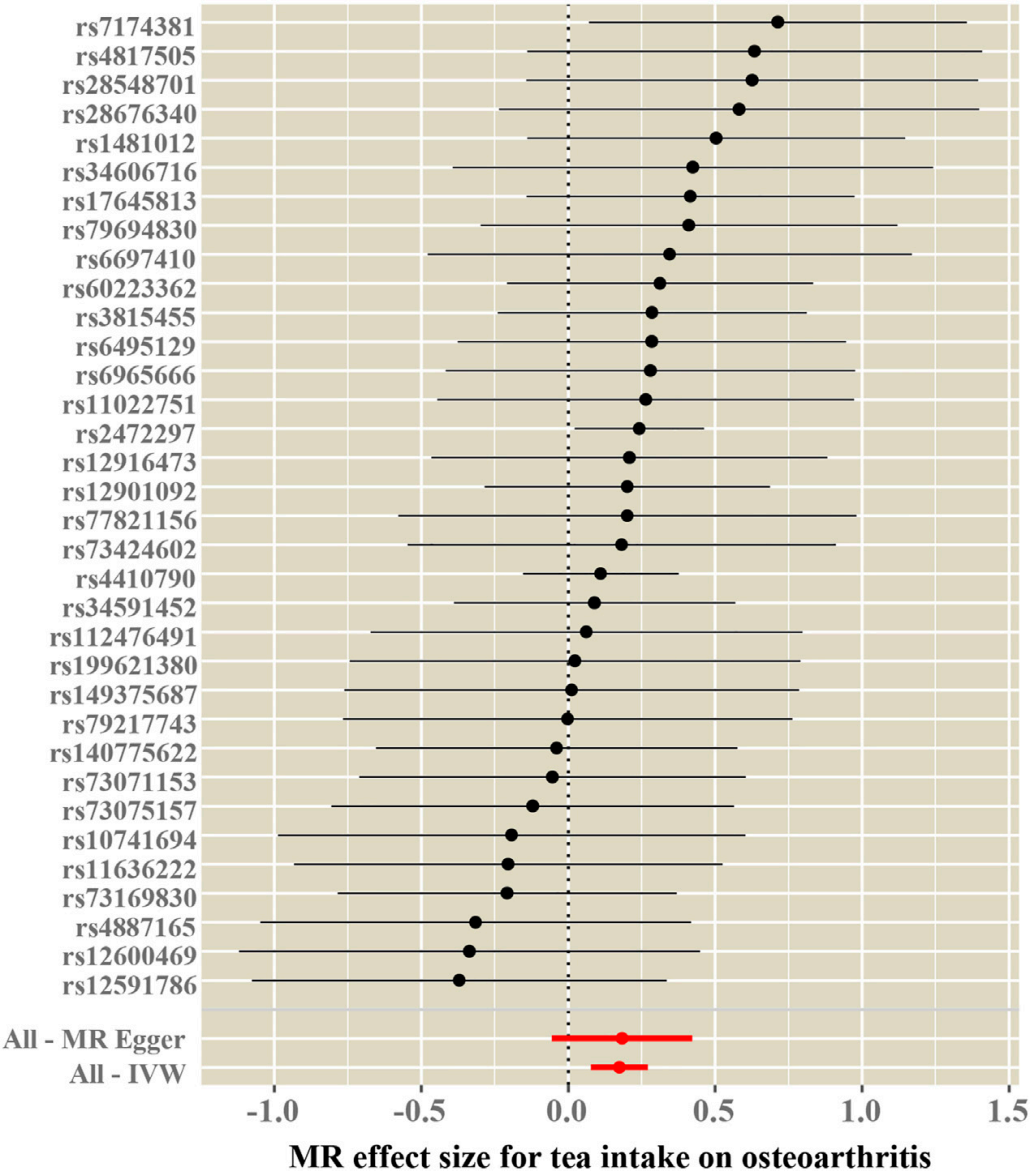
Forest plot of the effect size for each SNP on tea consumption and OA.

Cochran’s Q test and funnel plot did not indicate heterogeneity between SNP estimates based on single variants (Table 2; Figure 3). In the leave-one-out analysis, no SNP was found to be a potential outlier that could influence point estimates (Figure 4). In addition, the forest plot of each SNP-associated tea intake on OA showed that the majority of SNPs were in accordance with the same direction (Figure 5).

## Discussion

In recent years, aging has become increasingly pronounced globally. As a condition closely related to aging, the prevalence of OA faces an upward trend, placing heavy pressure on the healthcare system and patients’ quality of life. Tea intake may be associated with the incidence of OA, but the conclusions from animal models and clinical studies vary (Luk et al., 2020). Our study is the first to adopt MR to examine the causal link between tea intake and OA to provide new evidence in clinical settings.

Our results showed that tea intake increases the OA risk, which is inconsistent with previous reports. Epigallocatechin gallate (EGCG) is the major bioactive component of polyphenolic fraction in green tea. In cellular studies, Huang et al. (2015) used EGCG to treat primary rabbit articular chondrocytes and found that the EGCG-treated group had higher amounts of cartilage extracellular matrix and collagen II synthesized than the control group. Thus, EGCG may protect the cartilage from degradation and prevent OA. Similar results were reported by Elder et al. (2017) and Bae et al. (2010). This protective phenomenon is also replicated in animal models. Haqqi et al. (1999) reported that tea extracts and polyphenolic fraction could alleviate collagen-induced arthritis in mice. However, few studies were performed in humans, and their conclusions vary. In an open-label clinical study with 40 knee OA cases, patients treated with green tea extract for 4 weeks showed alleviated pain as assessed by the Visual Analog Scale but had the same extent of stiffness as assessed by WOMAC (Hashempur et al., 2018). Among the 40 participants, 80% were males. Considering the small number and uneven baseline population, the robustness of results may be careful. As a covariate in another study that enrolled 11,091 participants, the protective effects of green tea intake were observed in males but not in females (Takiguchi et al., 2019). In a further subgroup analysis, the protective effects disappeared in males aged more than 60 years, who are the high-risk population of OA. This preliminary finding was deduced based on observational study, which could not overcome endogeneity. By contrast, Chet et al. determined no causal association between tea intake and OA (knee OA and hip OA) by using MR method (Chen et al., 2022). However, in their studies, the odds ratios were greater than 1.00 with marginal significance (1.11 for knee OA; 1.20 for hip OA), indicating the likelihood of less statistical power. Therefore, further studies with more statistical power are required.

The mechanisms of the relation of tea to OA remain unknown. In previous studies, EGCG is used to imitate the function of tea and further explore the specific molecular mechanisms in cellular studies and animal models (Leong et al., 2014; Rasheed et al., 2016; Jin et al., 2020). Leong et al. (Leong et al., 2014) found that EGCG-treated mice (25 mg kg^−1^ per day) had reduced expression of pro-inflammatory cytokines, namely, IL-1β and TNF-α. Pain symptoms were also alleviated compared with those in the control group. However, EGCG cannot be used as a substitute of tea in human study. In addition to EGCG, other major components, such as caffeine, may influence OA. In a recent review, Guillán comprehensively summarized the evidence regarding the relationship between caffeine and OA and concluded that caffeine increases the OA risk (Guillan-Fresco et al., 2020). The influence of other compounds may lead to different conclusion between our study and others.

MR is an effective method used to evaluate causal links because it can overcome endogeneity in observational studies. It can also be used to test the relationships between other risk factors, such as caffeine and OA (Lee, 2018). To the best of our knowledge, this study is the first to use MR to investigate the relationship between tea intake and OA. However, this study has some limitations. First, the SNPs used are from the European population, which may lead to bias. Further validations in different races should be made. Moreover, this study used green tea and black tea, whose chemical components vary. Consequently, their effects also differ. The exposure and outcome examples were obtained from UK Biobank; as such, sample overlap could lead to substantial bias and inflated Type 1 error (Burgess, Davies, Thompson). Therefore, sensitivity analyses should be conducted using fewer genetic variants with stronger power, and two-sample method can be performed in further causal research.

Currently, tea intake is prevalent among aged individuals (Tang et al., 2019). Given that increased risks were reported in our study, tea should be carefully used as a common supplement and alternative medicine.

## Conclusion

New causal evidence is provided for the association of genetically predicted tea consumption with increased risks of OA.

## Data Availability

Publicly available datasets were analyzed in this study. The names of the repository/repositories and accession number(s) can be found in the article/supplementary material.
